# Q&A: inbreeding and its implications for conservation

**DOI:** 10.1186/s12915-025-02386-6

**Published:** 2025-10-21

**Authors:** Bárbara R. Parreira, Patrícia Pečnerová, Laura Tensen, Sabhrina Gita Aninta, Anna Brüniche-Olsen, Anubhab Khan, Hernán E. Morales, Lauren M. Hennelly

**Affiliations:** 1 Globe Institute, University of Copenhagen, Copenhagen, Denmark; 2 Center for Chromosome Stability, Department of Cellular and Molecular Medicine, University of Copenhagen, Copenhagen, Denmark; 3 Department of Biology, University of Copenhagen, Copenhagen, Denmark; 4 Department of Biology, Lund University, Sölvegatan 37, 22362 Lund, Sweden; 5 Department of Biology, Section Ecology and Evolution, University of Copenhagen, Copenhagen, Denmark; 6 Department of Zoology, Centre for Ecological Genomics and Wildlife Conservation, University of Johannesburg, Johannesburg, South Africa; 7 Center for Macroecology, Evolution and Climate, Globe Institute, University of Copenhagen, Copenhagen, Denmark; 8 Center for Ecological Sciences, Indian Institute of Science, Bengaluru, India; 9 Center for Conservation Genomics, Smithsonian’s National Zoo and Conservation Biology Institute, Washington, DC USA

## Abstract

Inbreeding depression plays a role in the decline, endangerment, and extinction of small populations, and thus inbreeding has received much attention in conservation biology. The term inbreeding is used across distinct fields but sometimes with varying meanings. This inconsistency creates misunderstandings that complicate interpretation, hinder comparisons among studies, and obstruct effective communication of findings. In this piece, we clarify the conceptual foundations of inbreeding, showcase modern estimates, and discuss its significance and relation to classical definitions. When used together, these interpretations can offer a more comprehensive understanding of the genetic dynamics in small populations.

## What is inbreeding, and why does it matter in conservation?

Species become a matter of conservation concern when they experience rapid or sustained population declines. As populations shrink, they become vulnerable to a range of stochastic threats, including environmental, demographic, and genetic — a phenomenon described by the small-population paradigm. Inbreeding is a genetic extinction factor. It widely refers to the mating of related individuals, that is, those that share common ancestors, and in small populations, mating with relatives is more likely. Inbreeding may lead to inbreeding depression effects “(the reduced fitness of offspring, see below)”, reducing population fitness, viability, and increasing extinction risk. Thus, inbreeding is an important genetic factor in conservation because conservation mostly deals with small populations. Understanding the extent of inbreeding in endangered populations informs conservation actions such as genetic rescue, managed breeding, habitat connectivity, and the prioritization of at-risk populations.

## So, what does inbreeding mean?

Inbreeding broadly refers to mating between individuals with shared ancestry, a definition so general that it has led to multiple interpretations and confusion. This was highlighted in key publications such as “Inbreeding: One word, several meanings, much confusion” [1]. More recent reviews on inbreeding in wild populations have focused on three interpretations of inbreeding that are most commonly used in conservation: inbreeding as non-random mating, inbreeding due to population subdivision, and individual inbreeding (traditionally pedigree inbreeding). The concept of inbreeding was developed to describe how genotypic frequencies change due to mating between closely related individuals — that is, individuals that are more closely related than two individuals chosen at random [2]. In its original formulation, inbreeding was quantified by the *F*_IS_ coefficient, which measures deviations from Hardy–Weinberg expectations within populations — inbreeding as non-random mating. Later, the concept was expanded to encompass the correlation of alleles due to population structure — inbreeding due to population subdivision. In this context, inbreeding arises from population subdivision: when a population is divided into smaller populations (demes), individuals within the same deme are, on average, more genetically similar to each other than to individuals from other demes. As a result, random matings within demes will often occur between genetically similar individuals. This broader interpretation is captured by the *F*-statistics framework, where *F*_ST_ and *F*_IT_ complement* F*_IS_ in describing genetic variation at different hierarchical levels. The last definition of inbreeding — individual inbreeding **— **estimates the proportion of the genome that is in identity-by-descent (IBD), as introduced by Malécot [3]. Here, inbreeding reflects the likelihood that an individual’s two homologous gene copies originate from the same ancestor. Given sufficient time, all alleles coalesce to common ancestors, and the probability of IBD increases by drift, even in randomly mating populations.

The persistent confusion between inbreeding as a deviation from random mating and inbreeding as IBD remains one of the major sources of misunderstanding in both theoretical and applied conservation genetics. This has important implications for how inbreeding is assessed and managed; most notably, that different measures of inbreeding are not directly comparable, and inbreeding is not always a consequence of a population decline.

## Can inbreeding be directly linked to fitness effects?

In plant and animal species, inbreeding has been consistently shown to reduce offspring survival and reproductive success [4]. This relationship — increased inbreeding and reduced fitness — is known as inbreeding depression and reflects the negative fitness consequences of increased homozygosity. While inbreeding depression is well documented in controlled breeding experiments, directly and quantitatively linking inbreeding as a cause of reduced fitness in wild populations remains a significant challenge [5]. This limitation rests on the complexity of measuring fitness in wild populations [6]. First, fitness itself is difficult to define: in short-lived species or experimental settings, it often refers to an individual’s survival, lifetime reproductive success, or viable offspring, but in wild populations, fitness more often refers to juvenile survival, mating success, or recruitment into the breeding population. This reflects practical constraints in the wild, as measuring fitness requires continued investment of resources into collecting long-term data. Therefore, studies investigating inbreeding depression tend to use fitness-related proxies that vary in assumptions about their direct relationship to fitness. Studies have found a correlation between inbreeding and such fitness-related proxies such as birth weight, juvenile survival, annual breeding success, and per capita population growth rate, using long-term monitored wild populations. A second challenge is that fitness-related metrics in wild populations are influenced by many factors, such as environmental and resource variability across space and time. Accounting for these confounding factors remains challenging due to the additional need to measure spatiotemporal environmental and ecological data.

## How can we assess inbreeding depression from genetic data?

**Fig. 1:**
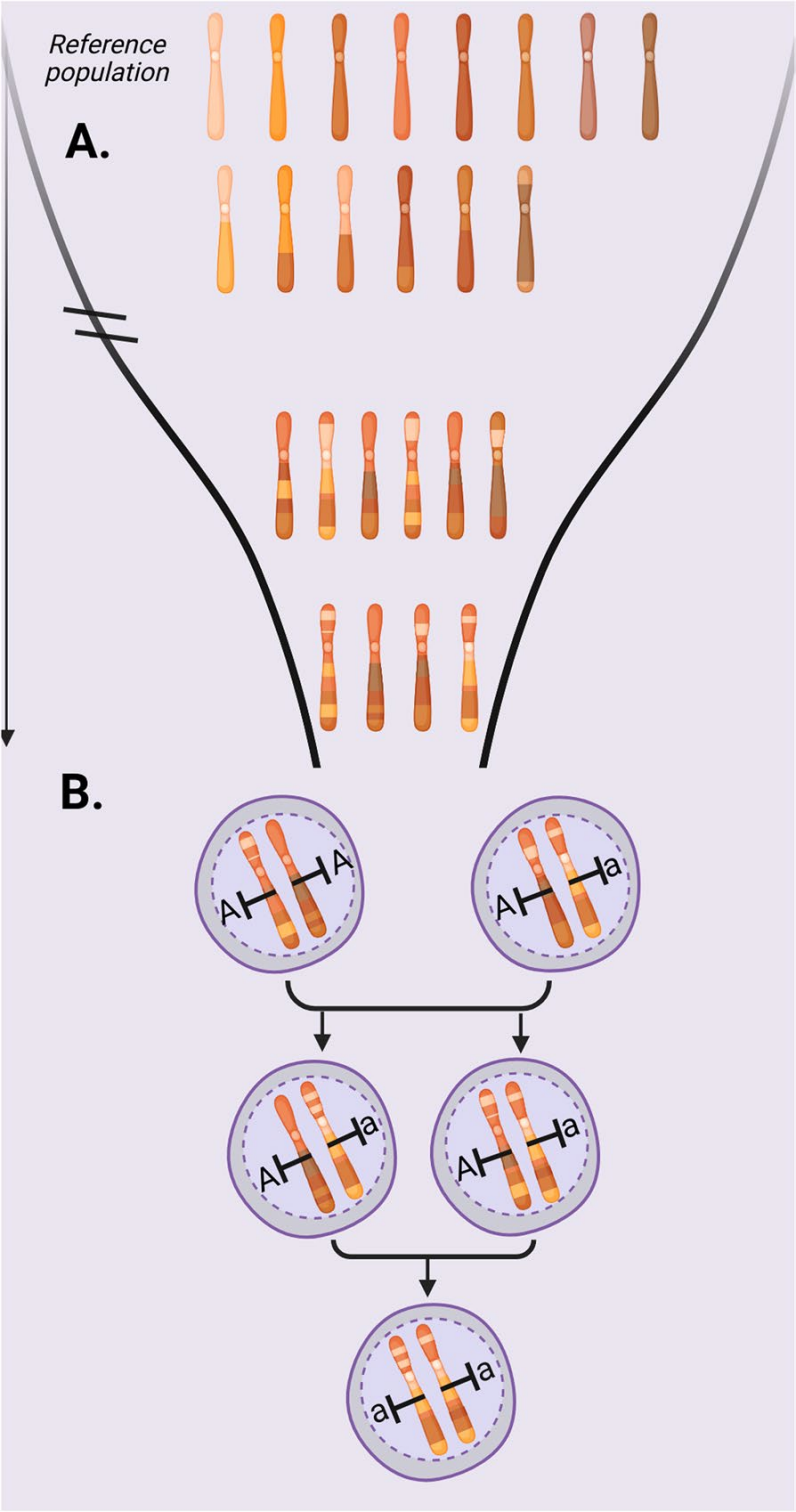
Illustration of identity-by-descent and deleterious homozygosity. **A** Identity-by-descent (IBD) is defined relative to a base population in which individuals are assumed to be unrelated — here represented by chromosomes composed of uniquely colored segments. Segments are IBD (same-colored regions) because they were inherited from a common ancestor without recombination. **B** The figure illustrates how, in a small population, random mates share a large portion of their genomes due to recent common ancestry—most genomic segments trace back to ancestors present at the time of the bottleneck. Small populations elevate the risk of recessive deleterious mutations becoming homozygous and thus, expressed. As a result, offspring inherit large, identical segments of DNA, leading to increased homozygosity — long stretches of the genome where both chromosomes carry the same alleles

Assessing inbreeding depression directly from genetic data relies on identifying and quantifying genetic variants that may reduce fitness. But the genetic underpinnings of inbreeding depression are largely unknown, as specific genes and pathways involved have yet to be identified, and these may vary among species and populations. Recent genomic approaches focus on identifying deleterious mutations, i.e., variants predicted to disrupt gene function. By summing the presumed harmful effects of these mutations across an individual’s genome, it becomes possible to approximate the genetic load, defined as the cumulative impact of deleterious mutations on fitness [7]. Although genetic load provides a conceptual link to inbreeding depression, estimating its real impact in natural populations remains challenging because the accumulation of load depends on specific demographic histories and local selection pressures [7, 8]. A complementary strategy is to correlate genomic measures of inbreeding “(e.g., *F*_ROH_, see below)” with genetic load estimates and/or fitness-related proxies. In theory, short runs of homozygosity (ROH) reflect tracts of old shared ancestry (i.e., background relatedness). In contrast, in longer ROHs (that have had fewer generations of recombination), purifying selection has had less time to remove highly deleterious mutations. As a result, these longer tracts tend to be more strongly associated with fitness declines (Fig. 1). Nevertheless, *F*_ROH_ alone does not reveal the extent to which ROH regions impact fitness. Drawing a direct link between inbreeding and reduced fitness further requires empirical data on traits such as survival, reproduction, or growth rates.

## Are there exceptions to general trends about inbreeding and fitness?

High levels of inbreeding have been shown to reduce fitness in wild and laboratory settings [4]. Still, some populations are highly inbred yet do not show signs of inbreeding depression (e.g., vaquita *Phocoena sinus*, the Chatham Island black robin *Petroica traversi* and Island foxes *Urocyon littoralis*, among many others). This apparent resilience to population decline likely results from a complex interplay between selection and past demographic history [7, 8]. In the face of population decline, the burden of deleterious variation is influenced by the ancestral population size. Ancestral large populations harbor a number of deleterious mutations in the heterozygous state as standing variation. Following a population collapse, this hidden load becomes exposed through increased homozygosity, leading to inbreeding depression. In contrast, long-term small populations hold deleterious mutations that tend to become homozygous more often. This increased homozygosity reveals their harmful effects on fitness, enabling natural selection to act more effectively against them, especially in the case of highly deleterious mutations (Fig. 1). Regardless of purging in long-term small populations, deleterious mutations will accumulate over time, particularly mildly deleterious ones, because selection is less effective in small populations. Thus, an apparent resilience to inbreeding depression depends on a balance between the purging and accumulation of deleterious variation. Moreover, many populations that have been persisting in low numbers live in habitats that have been stable over long periods of time. Even though these populations may not show inbreeding depression effects, they are still a matter of concern because they hold low genetic diversity — crucial to resist and adapt to future environmental changes. Another concern is that environmental changes can alter selective pressures, causing previously neutral or nearly neutral variants to become deleterious, placing even greater pressure on the already limited genetic diversity.

Finally, inbreeding varies significantly across species. The same inbreeding level may appear as high for one species but not necessarily for another. Specifically, larger-bodied species with longer generation times and lower fecundity tend to show higher inbreeding (*F*_ROH_) than smaller-bodied species, and carnivores exhibit higher *F*_*ROH*_ than herbivores [9]. Thus, some species appear to be more tolerant to inbreeding simply due to their life-history traits.

## Is inbreeding the same as identity by descent?

No, it is not. Measures of inbreeding ultimately relate to IBD but do not directly measure IBD. Inbreeding coefficients calculate the increase in the probability of IBD with respect to a reference base population. This reference population is always arbitrary. The different inbreeding estimates differ in what they use as the baseline population for the increase in IBD probability. For instance, when measured from pedigrees, inbreeding is measured relative to a founder population assumed as non-inbred, i.e., non-IBD. When calculated from genome-wide data, the arbitrary baseline against which inbreeding is measured can be given by different approaches. In inbreeding (i) as a measure of deviation from random-mating, the baseline is the expected heterozygosity in a hypothetical random-mating population and (ii) in inbreeding as a measure of individual inbreeding, the baseline is relative to a time limit that puts a background value on the depth of coalescence beyond which genes are assumed non-IBD. Inbreeding is thus a relative measure, given by an increase in the probability of IBD, but it is not equivalent to IBD [10].

## How can inbreeding be measured?

Quantifying inbreeding has been a key objective in many conservation genetics studies. This aim is not simply to gauge inbreeding levels, but ultimately to measure fitness, which is expected to decline due to inbreeding depression [6]. In practice, inbreeding can be quantified using empirical data from pedigrees, a few hundred genetic markers across many individuals, or genomic data that spans the entire genome. Genomic *F* estimators are pivotal tools in genetic research and are categorically divided into two distinct classes that reflect how they are calculated. The first class leverages correlations between single nucleotide polymorphisms (SNPs) to estimate inbreeding levels. *F*_UNI_ is based on the correlation between uniting gametes and compares observed homozygosity to the expected homozygosity under Hardy–Weinberg equilibrium, using allele frequencies from a reference population. *F*_GMR_ is also based on genome-wide SNP data — derived from the genomic relationship matrix (GRM) — and although it is an *F*-coefficient, it actually estimates relatedness (IBD sharing between individuals). The second class of coefficients of inbreeding are based on identifying homozygosity along the genome. It includes F_HOM_, an estimator based on the expected and observed individual heterozygosity (or homozygosity) and that is the genomics equivalent to Wright’s *F*_IS_ index. *F*_ROH_ identifies ROH along the genome and aims at reflecting the amount of IBD sharing within an individual. Finally, *F*_PED_, equivalent to *F*_ROH_ but measured from pedigree data, is calculated by tracing the path between two gene copies within an individual through its parents to a common ancestor to estimate the proportion of shared ancestry between the parents.

The comparison between the different inbreeding coefficients has been quantified based on theoretical properties, such as bias and standard errors, comparisons among the different inbreeding coefficients to pedigree-based estimates, or evaluating which estimates show the highest correlation to fitness [11]. Yet, there is no universal agreement on which estimator is the best. Note that different *F* estimators also differ in what aspects of inbreeding they emphasize and are thus not directly comparable: *F*_HOM_ and *F*_UNI_ measure inbreeding as non-random mating, and *F*_PED_ and *F*_ROH_ measure inbreeding as a consequence of genetic drift.

## What do inbreeding measures really tell us?

Inbreeding is a result of life-history, behavioural, and demographic factors, such as population structure or past fluctuations in structure and size. As such, inbreeding measures without proper context may be misleading or meaningless. For instance, deviations from random mating can result from hidden population structure that causes an overall deficiency of heterozygosity (*F*_IS_ > 0), and the magnitude of this deviation depends on factors like the number of demes, divergence time, and gene flow. On the other hand, random fluctuations in allele frequencies that occur as a consequence of reproduction in small populations can lead to an excess of heterozygotes. This excess is particularly expected in species with high sex bias due to binomial sampling error occurring among the sexes.

Social structure also influences inbreeding. Theoretical and simulation works accounting for subdivision into breeding groups have shown that social structure can lead to an excess of heterozygotes without explicit inbreeding-avoidance strategies [12]. *F*_IS_ is expected to be negative independently of the breeding and dispersal behaviour and is expected to be maximized when groups are small, polygyny is high, and parents differ in origin. This is particularly important in the context of behavioural ecology studies, where deviations from random mating are often interpreted as indicators of evidence for inbreeding avoidance strategies. However, it is crucial to note that the inbreeding coefficients just reflect an increase or decrease in inbred relationships and do not offer insights into behavioral mechanisms. Thus, while important and relevant for conservation, inbreeding measures should be critically analyzed, taking into consideration their underlying assumptions, methodological scope, and interpretative limitations.

## What natural mechanisms minimize “inbreeding”?

The *F* inbreeding coefficients merely quantify inbreeding but do not reveal its underlying causes. Yet, many studies discuss the adaptive value of inbreeding avoidance, and numerous behavioral mechanisms are interpreted as strategies to minimize inbreeding. Mating bias against relatives, sex-biased dispersal, or post-copulatory mechanisms such as sperm rejection, sperm selection, and biased fertility are usually considered inbreeding avoidance strategies. They appear to be more pronounced in monogamous species, *K*-selected species (which have small offspring numbers), and species with small populations, where kinship encounters are frequent. Although there is evidence that animals may indeed be able to recognize kinship through phenotype matching, familiarity during early life, or olfactory receptor genes, this too remains controversial. Moreover, some argue that inbreeding avoidance theory overlooks inbreeding tolerance or preference, which is well documented in many species [13]. Such studies highlight potential benefits of inbreeding, such as increased ability of parents to spread their genes (e.g., inclusive fitness). These are argued to outweigh the costs of inbreeding, especially when consanguineous matings occur among relatives other than siblings. Presumed inbreeding avoidance mechanisms may influence genetic structure, yet *F* coefficients alone can not reveal their presence.

## What are the future directions for studying inbreeding in conservation?

In today’s biodiversity crisis, more species are confined to increasingly smaller and isolated populations. Therefore, quantifying inbreeding and its effect on fitness has a high priority in conservation. Still, a mechanistic understanding of inbreeding and fitness remains a challenge. This is due to several reasons. Most threatened species lack high-quality reference genomes, limiting our ability to assess ROH and describe overarching patterns across species. More importantly, microsatellites, still the most used marker in conservation genetics, can measure deviations from random-mating or population subdivision, but cannot measure individual inbreeding. Current methods to identify deleterious variants rely on numerous assumptions, are rarely validated, and are often focused on protein-coding regions of the genome. Machine learning is improving our ability to assess the functional consequences of mutations, such as by predicting regulatory regions and 3D protein structures to estimate a mutation’s functional impact on fitness [14]. However, the lack of sufficient training data from non-model species and the challenges of validating predictions in real-world settings remain significant obstacles. Integrating insights from livestock, plant, and human populations, where inbreeding research is well-developed, could further enhance our understanding of the genetics of inbreeding depression in wild populations 

Using genetic data to quantify inbreeding depression extends beyond deleterious mutations and encompasses other dimensions of the genome, such as epigenetic modifications. For instance, in the plant *Scabiosa columbaria*, inbred individuals exhibited higher levels of DNA methylation compared to outbred ones [15]. Remarkably, treatments to reduce methylation significantly alleviate inbreeding depression. These findings challenge the conventional view that inbreeding depression is solely driven by the accumulation of deleterious mutations, highlighting the potential role of epigenetic mechanisms.

Finally, genetics is only one component to understand inbreeding and its impact on fitness. Fully understanding the conservation implications of inbreeding depends on many factors we cannot measure from the genome: animal behavior, environmental stochasticity, density dependence, and resource availability can all influence the extent at which inbreeding occurs, how inbreeding depression manifests, and how quickly populations can recover. Future work that integrates demographic, (epi)genetic, ecological, and environmental data will allow a better understanding of inbreeding and its role in conservation.

## Data Availability

No datasets were generated or analysed during the current study.
